# Where Does Your Soul Find Meaning? Reflections of a Cancer-Experienced Physician on Meaning in Medical Education

**DOI:** 10.5334/pme.2888

**Published:** 2026-10-28

**Authors:** Sandra Apondo

**Affiliations:** 1 TUM Medical Education Center, TUM School of Medicine and Health, Technical University Munich, Germany

## Abstract

Drawing on experiences as a medical student, simulated patient, physician, cancer patient, and medical educator, this reflective essay explores the search for meaning within medicine and within one’s own life. Moving between illness, care, teaching, vulnerability, and encounters with mortality, the text reflects on how experiences within medicine shape professional identity, personal values, and the understanding of what it means to care for another human being. The essay invites readers to pause and reflect on where meaning, connection, and inner orientation can be found in both professional and personal life.

Over nearly two decades, I have moved through medicine in many different roles: as a student and simulated patient (SP), physician and patient, and finally educator and researcher. Looking back, I realize that these experiences did not simply accumulate but transformed one another. Each reshaped the meaning of those that came before and those that followed. Time and again, I found myself encountering the same questions in new forms: What does it mean to care not only for the body, but also for the soul? What gives human life meaning? The reflections that follow describe not only moments from a personal journey but also the gradual formation—and transformation—of my identity as a medical educator teaching future physicians.

## Medical Student

Dusanka liked it when I recited the Lord’s Prayer to her, often several times in a row. A progressive neurological disease had first paralyzed her entire body and almost stolen her voice. We communicated through glances, the laying on of a hand, a smile, or tears. For many weeks, three friends and I accompanied Dusanka, who had no relatives nearby, on her final journey.

**Figure:**
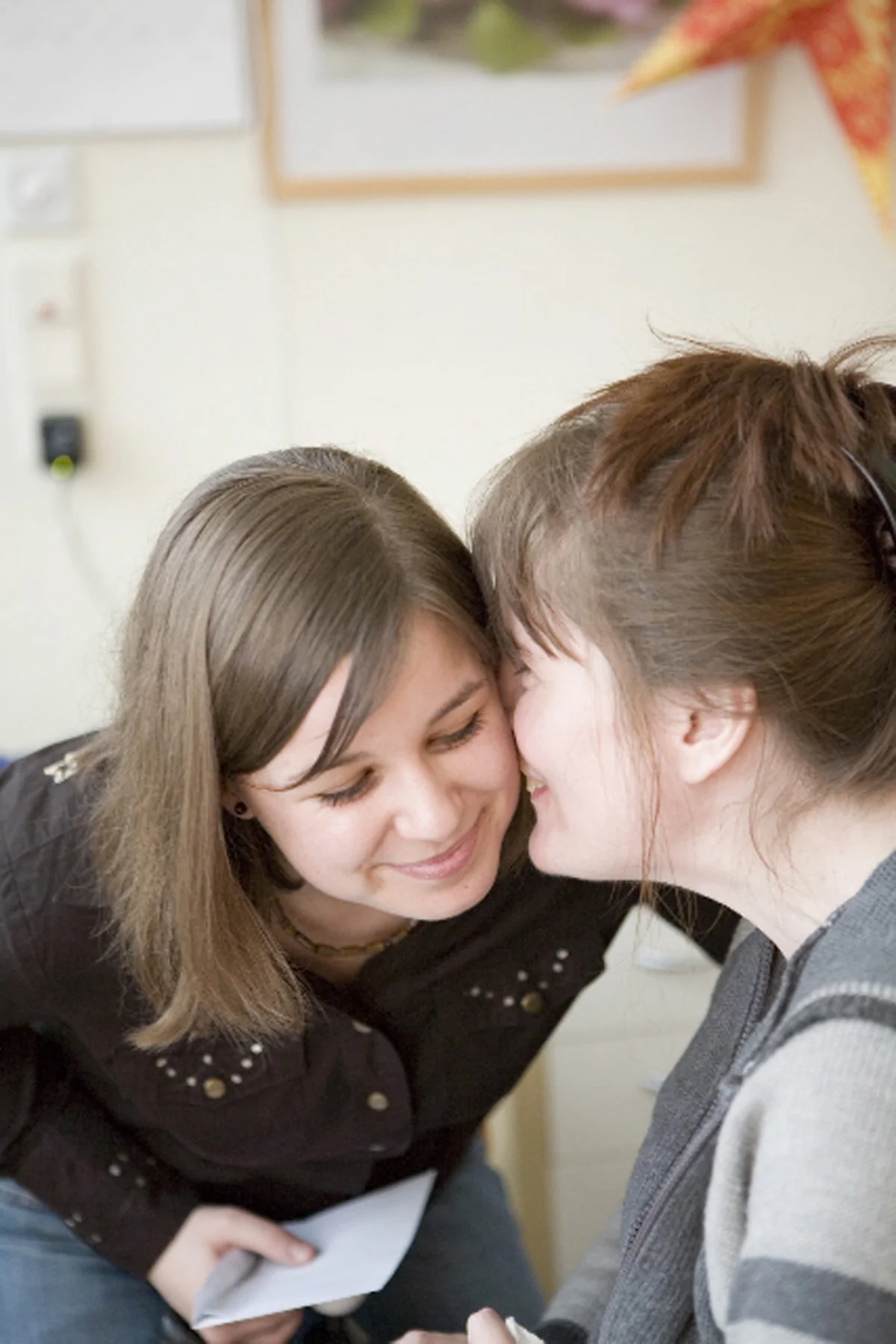
Dusanka (right), Sandra (left), 2008.

Then 22, I rushed between medical lectures, choir rehearsals, church services, and Dusanka’s hospice. Sometimes I slept in the armchair beside her bed, sometimes during class. I had known Dusanka for about eight months, and throughout that time she was dying; she lived and laughed and yet was dying. Every day her 35-year-old body came a little closer to death, while Dusanka taught me more about my own life. Being with her, singing Brahms’ Requiem or reading Foucault gave my life more meaning than pipetting in the biochemistry lab. At the time, I believed I was accompanying Dusanka through the end of her life. Only much later did I realize that she had been accompanying me through the beginning of my own. She shared the moment of her death with me on a hot early-summer afternoon in 2008, when I once again skipped a lecture in order to be with her. I went on and on repeating the Lord’s Prayer to her, and maybe more so to myself. Her thin fingers lay limp in my strong hand, a hand accustomed to lifting kettlebells in the gym and striking décimes on the piano. While my life was beginning to blossom, hers was withering away. And then she took her last breath.

## Simulated Patient

Around 2011 as a medical student in a German university town like Heidelberg, where partying was practically a way of life, one always needed money. I earned mine through my fees as a simulated patient (SP). My favourite role: “Ms. K.,” a 25-year-old law student with relapsed Acute Myeloid Leukemia (AML) in a palliative situation. The medical students — my fellow students one year below me — were supposed to “break the bad news” to me: “You are going to die.” Some of them did this well as they handed me tissues for the artificial tears running down my pale, grey-painted face. I loved playing this role and found it beautiful, because it was about something existential, not merely a sprained ankle. This acting was about what already felt real to me. Death is always part of even the most blossoming, youthful life — that is what Dusanka taught me.

A few years later, when illness entered my own family—and eventually my own body—I began to understand why this role had always felt strangely familiar. In 2016, my 70-year-old Romanian grandmother was diagnosed with AML. AML was now no longer part of my script with artificial tears, but part of my reality with real tears. Suddenly, the role I had once played became my own story. From the very beginning, nothing could be done. Death claimed her quickly, less than two weeks after she and my then one-year-old son — her first great-grandchild — shuffled hand in hand down the shabby hallway of a small Romanian hospital, unclear who was holding on to whom. It was Christmas 2016 and the hospital smelled of home-cooked food brought in by the patients’ relatives. In the hallway stood blinking plastic Christmas trees beside statues of the Virgin Mary next to Orthodox icons. Some of the weary patients paused to cross themselves before these religious images, as if to find a moment of inner steadiness. Others passed them by, sunk into themselves, perhaps more connected with their souls than we could guess.

## Physician

The soul — that was my thing, though somehow, it eluded me during my medical studies. Certainly, I could not find it in my clumsy attempts at suturing bananas, or in pressing on mannequins to the rhythm of “Staying Alive,” meant to prepare us for our first real resuscitation.

Dusanka on the other hand, allowed me to look into her gentle soul. How she managed that with her few single words stretched over several seconds, I do not know. But she did. Like all the hospice staff back then, I was certain that her soul escaped from her dead, cooling body through the open window into the Heidelberg summer heat.

Fortunately, I encountered many other souls in real life. As a psychiatrist, I wanted to care for people whose lives had become torment through depression, psychosis, or anxiety. I tried to understand their suffering as a physician. I would soon discover how incomplete that understanding was. I was able to accompany some patients for part of their journey. Some recovered or at least became a little less ill. Others, despite all our efforts, suffered such profound anguish that they ended their own lives hoping to let their souls escape.

## Patient

2021, after eight years working in psychiatry, now with two children of kindergarten age, I found myself shuffling down the sterile university hospital hallways, hung with abstract art— bald, my swollen hands clinging to the chemo – IV pole, my constant companion.

In my first real breaking bad news conversation I was told: “PMBCL (Primary Mediastinal Large B-Cell Lymphoma), an aggressive lymphoma, but highly curable. The prognosis is excellent.” Not a word about dying, unlike back then when my simulated AML-SP-self was told: “You are going to die.” The irony returned once again: I had “had” AML as an SP, my grandmother died of AML, I do have PMBCL. How had I slipped into this hemato-oncology drawer and got stuck there?

During many weeks of lying awake in hospital beds, assailed by relentless thoughts and physical pain (for which, thankfully, there is very effective morphine), I came closer to my soul than in hundreds of hours of psychotherapeutic self-exploration as a psychiatrist. I discovered unpleasant things there too: disorder, unlived life. Certainly not a life ready for death.

Of course, the rational physician within me knew: “You are not going to die.” The oncologists told me countless times: “This is not about dying. You will almost certainly recover.” And they were right. But even the most optimistic person thinks about death when waking, drenched in sweat, nauseous, feverish, unable to eat solids, able only to drink coke. You still think this could be the end.

You fall asleep eventually in the bed where maybe someone before you has died or someone after you will die in. You are woken up by the nurse’s cheerful “Good morning” at 7am. She opens the window saying: “You need fresh air”. Gentle sunlight streams into the room. I wonder whether some of my worries have just escaped through the window and dissolved into the mild summer breeze. I tell myself: my soul stays right here with me. It is not allowed to leave yet.

## Medical Educator

My doctors were right. Five years later, in 2026, I am joyfully alive. Over time, I came to realize that learning medicine as a student, practising it as a physician, and experiencing it from the other side as a cancer patient shaped the educator I have become. As a curriculum developer, I now write the role scripts for our SPs myself. Breaking bad news conversations are not meant merely to be performed correctly, but to address social and psychological dimensions as well. In short, students should learn to address the whole human being, not only their body, but also their soul.

We demand a great deal from our students. Today, they train in high tech simulation operating rooms. We can let the mannequin die at the push of a button, the monitor flatlines, the students stop pushing to the “Staying Alive” beats and gather for a debriefing. There is no real death, and therefore no soul escaping into the air (there aren’t any windows either, in the underground-built simulated OR).

What does exist, however, are the young souls of these students: one may be depressed, one happily infatuated, another grieving, another longing for connection. We rarely know what is truly happening within them and what gives their souls meaning. Or as Viktor E. Frankl wrote: “Meaning is the home of the soul” (Der Seele Heimat ist der Sinn) [1]. Where students find that home, we as teachers do not know. But we could ask. Not all the hundreds of students passing through our courses each semester, but perhaps one or two. We could simply say: “You seem thoughtful. How are you?” That would mean that we care for their souls as well as their education.

We expect our students to recognize that every patient is more than a suffering body. We must teach them that the soul of the sick person can be much more fragile or on the contrary, much stronger, than even the sickest body. The same is true for us physicians and teachers in our, mainly quite healthy bodies: our souls can, sometimes or often, feel very vulnerable. I believe that soul care should begin much earlier in medical education: in the anatomy lab, during bedside teaching, in simulation-based training, in the OR — wherever medical students encounter other human beings, patients or colleagues. Such encounters may become sources of meaning — for all of us alike.
